# Supplementary material for: Cost-effectiveness of alternative minimum recall intervals between whole blood donations
Source: PLoS One. 2022 Aug 17;17(8):e0272854. doi: 10.1371/journal.pone.0272854 (PMC9384981; doi:10.1371/journal.pone.0272854)
Supplement: S1 Appendix — (DOCX) [file pone.0272854.s001.docx]

# Appendix Table 1. Unit costs

| **Resource-generating event** | **Unit cost, £** | **Main source** |
| --- | --- | --- |
| 3-stage reminder for invitation to donate | 2.68 | Expert opinion |
| Low Hb deferral, additional costs at donor centre | 4.78 | Expert opinion |
| Low Hb deferral, subsequent health care costs | 5.39 | Expert opinion |
| Deferral due to other reasons | 0.97 | Expert opinion |
| 3-stage reminder following non-attendance | 3.10 | Expert opinion |
| Fainting episode at blood donation visit | 20.23 | Expert opinion |
| Variable cost of collecting 1 unit blood (centre operating with capacity) | 7.62 | NHSBT |

# Appendix Table 2: Number (%) of responses to the SF-36/SF-12 questionnaire at each timepoint

|  | **Randomised arm (male)** | | | **Randomised arm (female)** | | |
| --- | --- | --- | --- | --- | --- | --- |
|  | **8-wk**  **(n=7,417)** | **10-wk**  **(n=7,413)** | **12-wk**  **(n=7,411)** | **12-wk**  **(n=7,549)** | **14-wk**  **(n=7,545)** | **16-wk**  **(n=7,528)** |
| **Baseline** | 6,766 (91) | 6,765 (91) | 6,778 (91) | 6,776 (90) | 6,724 (89) | 6,746 (90) |
| **6 months** | 5,714 (77) | 5,644 (76) | 5,527 (75) | 5,673 (75) | 5,760 (76) | 5,652 (75) |
| **12 months** | 5,197 (70) | 5,183 (70) | 5,162 (70) | 5,148 (68) | 5,210 (69) | 5,133 (68) |
| **18 months** | 4,633 (62) | 4,659 (63) | 4,679 (63) | 4,515 (60) | 4,624 (61) | 4,533 (60) |
| **24 months** | 4,701 (63) | 4,747 (64) | 4,746 (64) | 4,506 (60) | 4,486 (59) | 4,444 (59) |

Results are presented for the SF-36/SF-12 questionnaires with complete information to calculate the SF-6D score.

**Appendix Table 3a. Results from logistic regression model for deferral for low Hb and deferral for other reasons for males**

|  | **Deferral for low Hb** | | | **Deferral for other reasons** | | |
| --- | --- | --- | --- | --- | --- | --- |
|  | **Odds ratio** | **SE** | **p-value** | **Odds ratio** | **SE** | **p-value** |
| Randomised arm: 8-week | 2.312 | 0.099 | 0.000 | 0.909 | 0.029 | 0.003 |
| Randomised arm: 10-week | 1.479 | 0.069 | 0.000 | 0.954 | 0.032 | 0.153 |
| Randomised arm: 12-week | 2.312 | 0.099 | 0.000 | 0.050 | 0.001 | 0.000 |
| Log-likelihood | -25519.042 | | | -27698.956 | | |
| Wald chi2 | 437.81 | | | 8.69 | | |
| Prob > chi2 | 0.000 | | | 0.013 | | |

**Appendix Table 3b. Results from logistic regression model for deferral for low Hb and deferral for other reasons for females**

|  | **Deferral for low Hb** | | | **Deferral for other reasons** | | |
| --- | --- | --- | --- | --- | --- | --- |
|  | **Odds ratio** | **SE** | **p-value** | **Odds ratio** | **SE** | **p-value** |
| Randomised arm: 12-week | 1.615 | 0.061 | 0.000 | 0.896 | 0.028 | 0.001 |
| Randomised arm: 14-week | 1.333 | 0.054 | 0.000 | 0.953 | 0.030 | 0.130 |
| Randomised arm: 16-week | 0.053 | 0.002 | 0.000 | 0.078 | 0.002 | 0.000 |
| Log-likelihood | -25198.765 | | | -26011.912 | | |
| Wald chi2 | 161.72 | | | 12.17 | | |
| Prob > chi2 | 0.000 | | | 0.002 | | |

# Appendix Table 4. Other health care events over two-year follow-up period, by randomised arm and gender

|  | **Randomised arm (male)** | | | **Randomised arm (female)** | | | |
| --- | --- | --- | --- | --- | --- | --- | --- |
|  | **8-week**  **(n=7,417)** | **10-week**  **(n=7,413)** | **12-week**  **(n=7,411)** | | **12-week**  **(n=7,549)** | **14-week**  **(n=7,545)** | **16-week**  **(n=7,528)** |
| **N (%) any serious adverse event** | 284  (3.83) | 257  (3.47) | 267  (3.60) | | 290  (3.84) | 289  (3.83) | 288  (3.83) |
| **N (%) doctor confirmed heart problems** | 25  (0.34) | 36  (0.49) | 21  (0.28) | | 5  (0.07) | 3  (0.04) | 12  (0.16) |
| **N (%) been to hospital after a fall** | 172  (2.32) | 130  (1.75) | 149  (2.01) | | 230  (3.05) | 232  (3.07) | 226  (3.00) |
| **N (%) been to hospital after a transport accident** | 104  (1.40) | 110  (1.48) | 115  (1.55) | | 66  (0.87) | 63  (0.83) | 61  (0.81) |

**Appendix Table 5a. Results from Generalised Estimating Equation (GEE) regression model for SF-6D score for males**

|  | **Coefficient** | **Standard error** | **p-value** |
| --- | --- | --- | --- |
| **Randomised arm*** |  |  |  |
| 8-week | 0.001 | 0.001 | 0.579 |
| 10-week | 0.000 | 0.001 | 0.907 |
| **Month**** |  |  |  |
| 6-month | -0.009 | 0.001 | 0.000 |
| 12-month | -0.012 | 0.001 | 0.000 |
| 18-month | -0.014 | 0.001 | 0.000 |
| 24-month | -0.023 | 0.001 | 0.000 |
| **Randomised arm * month** |  |  |  |
| 8-week*6-month | 0.001 | 0.002 | 0.707 |
| 8-week*12-month | -0.002 | 0.002 | 0.354 |
| 8-week*18-month | -0.001 | 0.002 | 0.720 |
| 8-week*24-month | 0.001 | 0.002 | 0.536 |
| 10-week*6-month | 0.000 | 0.002 | 0.811 |
| 10-week*12-month | 0.000 | 0.002 | 0.838 |
| 10-week*18-month | 0.000 | 0.002 | 0.868 |
| 10-week*24-month | -0.001 | 0.002 | 0.752 |
| **Constant** | 0.860 | 0.001 | 0.000 |
| Wald chi2 | 983.40 | | |
| Prob > chi2 | 0.000 | | |

* reference group - 12-week; ** reference group - baseline

**Appendix Table 5b. Results from Generalised Estimating Equation (GEE) regression model for SF-6D score for females**

|  | **Coefficient** | **Standard error** | **p-value** |
| --- | --- | --- | --- |
| **Randomised arm*** |  |  |  |
| 12-week | 0.001 | 0.001 | 0.594 |
| 14-week | 0.001 | 0.001 | 0.339 |
| **Month**** |  |  |  |
| 6-month | -0.020 | 0.001 | 0.000 |
| 12-month | -0.023 | 0.001 | 0.000 |
| 18-month | -0.026 | 0.002 | 0.000 |
| 24-month | -0.030 | 0.001 | 0.000 |
| **Randomised arm * month** |  |  |  |
| 12-week*6-month | 0.004 | 0.002 | 0.055 |
| 12-week*12-month | -0.001 | 0.002 | 0.787 |
| 12-week*18-month | -0.001 | 0.002 | 0.785 |
| 12-week*24-month | 0.000 | 0.002 | 0.838 |
| 14-week*6-month | 0.000 | 0.002 | 0.845 |
| 14-week*12-month | -0.001 | 0.002 | 0.679 |
| 14-week*18-month | 0.002 | 0.002 | 0.359 |
| 14-week*24-month | 0.002 | 0.002 | 0.383 |
| **Constant** | 0.847 | 0.001 | 0.000 |
| Wald chi2 | 1692.36 | | |
| Prob > chi2 | 0.000 | | |

* reference group - 16-week; ** reference group - baseline

# Appendix Table 6: Mean SF-6D score at each timepoint, by randomised arm and gender

|  | **Randomised arm (male)** | | | **Randomised arm (female)** | | |
| --- | --- | --- | --- | --- | --- | --- |
|  | **8-wk**  **(n=7,417)** | **10-wk**  **(n=7,413)** | **12-wk**  **(n=7,411)** | **12-wk**  **(n=7,549)** | **14-wk**  **(n=7,545)** | **16-wk**  **(n=7,528)** |
| **Baseline** | 0.86 | 0.86 | 0.86 | 0.85 | 0.85 | 0.85 |
| **6 months** | 0.85 | 0.85 | 0.85 | 0.83 | 0.83 | 0.83 |
| **12 months** | 0.85 | 0.85 | 0.85 | 0.82 | 0.82 | 0.82 |
| **18 months** | 0.85 | 0.85 | 0.85 | 0.82 | 0.82 | 0.82 |
| **24 months** | 0.84 | 0.84 | 0.84 | 0.82 | 0.82 | 0.82 |

**Appendix Table 7a. Results from Seemingly Unrelated Regression (SUR) model for males**

|  | **Costs** | | | **Number of donations** | | |
| --- | --- | --- | --- | --- | --- | --- |
|  | **Coefficient** | **Standard error** | **p-value** | **Coefficient** | **Standard error** | **p-value** |
| Rondomised arm: 8-week* | 16.171 | 0.402 | 0.000 | 1.701 | 0.052 | 0.000 |
| Rondomised arm: 10-week* | 7.089 | 0.402 | 0.000 | 0.788 | 0.052 | 0.000 |
| RMSE | 24.483 | | | 3.136 | | |
| R^2^ | 0.123 | | | 0.096 | | |

* reference group - 12-week

* Adjusted for age, ‘standard’ versus ‘high’ demand blood types, ethnicity, new donor or not, and recruitment source.

**Appendix Table 7b. Results from Seemingly Unrelated Regression (SUR) model for females**

|  | **Costs** | | | **Number of donations** | | |
| --- | --- | --- | --- | --- | --- | --- |
|  | **Coefficient** | **Standard error** | **p-value** | **Coefficient** | **Standard error** | **p-value** |
| Rondomised arm: 12-week* | 8.637 | 0.298 | 0.000 | 0.850 | 0.036 | 0.000 |
| Rondomised arm: 14-week* | 4.619 | 0.298 | 0.000 | 0.463 | 0.036 | 0.000 |
| RMSE | 18.263 | | | 2.224 | | |
| R^2^ | 0.116 | | | 0.128 | | |

* reference group - 16-week

* Adjusted for age, ‘standard’ versus ‘high’ demand blood types, ethnicity, new donor or not, and recruitment source.

**Appendix Figure 1. Uncertainty in the incremental costs (£ GBP) and number of whole blood donations, and their joint distribution, for reduced interval strategies versus standard practice (control arm) over two-year follow-up**

*a) Male*

*b) Female*
