# Supplementary material for: Cost-effectiveness of alternative minimum recall intervals between whole blood donations
Source: PLoS One. 2022 Aug 17;17(8):e0272854. doi: 10.1371/journal.pone.0272854 (PMC9384981; doi:10.1371/journal.pone.0272854)
Supplement: Attachment — Submitted filename: Response to Reviewers.docx [file pone.0272854.s002.docx]

Dear Editor,

Thank you for your useful comments as below. Please find my response as below.

We have noted that you have a related article published DOI 10.3310/hsdr06400 which addresses a similar research question with some overlap in the conclusions reported. Thank you for your reference of this article as ref. number 13, however please could you provide some additional feedback as to how the current manuscript further contributes to scientific knowledge in light of the related submission.

Please note that one of our publication criteria requires that "If a submitted study replicates or is very similar to previous work, authors must provide a sound scientific rationale for the submitted work and clearly reference and discuss the existing literature." ([https://journals.plos.org/plosone/s/criteria-for-publication#loc-2](https://protect-eu.mimecast.com/s/qQvsCyXjgiyp4JLhQHqK8?domain=journals.plos.org)).

***Response****: Reference 13 is the NIHR report and includes the findings of the cost-effectiveness analysis of the INTERVAL trial interventions. This submission is broader in scope than the cost-effectiveness analysis reported in reference 13. The main extension is that this submission considers a wide range of of policy relevant subgroups, whereas the subgroup results presented in Reference 13 were limited to those considered in the INTERVASL trial analysis. In particular, the current submission is motivated by the policy context in considering the relative cost-effectiveness of alternative interventions for those subgroups of donors whose blood type is in ‘high demand’. This implies that the introduction and discussion, which contextualises the important findings with respect to these donor subgroups are new and have not been published elsewhere. We have made the distinction between the material presented in this paper and the NIHR report plain in the revised submission.*

*Inevitably there is some overlap between the material presented in this paper, and the NIHR report (reference 13). We would like to highlight precedents where the main study report included all relevant aspects of the overall study including cost-effectiveness analysis, but that the cost-effectiveness analysis was also published as a standalone paper in a reputed journal. Here are a few examples.*

*1(a). Legood, R., Opondo, C., Warren, W., Jamal, F., Bonell, C., Viner, R., Sadique. Z. (2021), Cost-utility analysis of a complex intervention to reduce school-based bullying and aggression: an analysis of the INCLUSIVE RCT, Value in Health, 24(1):129-135.*

*1(b). Bonell, C., Allen, E., Warren, E., McGowan, J., Bevilacqua, L., Jamal, F., Sadique, Z., Legood, R., Wiggins, M., Opondo, C., Mathiot, A., Sturgess, J., Paparini, S., Fletcher, A., Perry, M., West, G., Tancred, T., Scott, S., Elbourne, D., Christie, D., Bond, L., Viner, R. M. (2019), Modifying the secondary school environment to reduce bullying and aggression: the INCLUSIVE cluster RCT. Public Health Research, NIHR Health Technology Assessment, 7(18): 1-164.*

*2(a). Sadique, Z., Harrison, DA., Bear, DE., Rowan, KM., Grieve, R., CALORIES trial investigators. (2018), Effectiveness and cost-effectiveness of early nutritional support via the parenteral versus the enteral route for critically ill adult patients. Journal of critical care.52, 237-241.*

*2(b). Harvey, SE., Parrott, F., Harrison, DA., Sadique, MZ., Grieve, RD., Canter, RR., et al. (2016), A multicentre, randomised controlled trial comparing the clinical effectiveness and cost-effectiveness of early nutritional support via the parenteral versus the enteral route in critically ill patients (CALORIES). Health Technology Assessment, 20(28):1-144.*

*3(a). Grieve, R., Sadique, Z., Gomes, M., Smith, M., Lecky, F., Hutchinson, P., Menon, DK., Rowan, K., Harrison, D. (2016), An evaluation of the clinical and cost-effectiveness of alternative care locations for critically ill adult patients with acute traumatic brain injury, British Journal of Neurosurgery, 30(4):388-96.*

*3(b). Harrison, D., Prabhu, G., Grieve, R., Harvey, SE., Sadique, MZ., Gomes, M., Griggs, KA., Walmsley, E., Smith, M., Yeoman, P., Lecky, FE., Hutchinson, PJA., Menon, D., and Rowan, KM. (2013), Risk Adjustment In Neurocritical care (RAIN): prospective validation of risk prediction models for adult patients with acute traumatic brain injury to use to evaluate the optimum location and comparative costs of neurocritical care, Health Technology Assessment, 17(3):1-350.*

*4(a). Sadique, Z., Grieve, R., Harrison, D.A., Jit, M., Allen, E., Rowan, K.M., FIRE Study Investigators. (2013), An integrated approach to evaluating alternative risk prediction strategies: a case study comparing alternative approaches for preventing invasive fungal disease, Value in Health, 16(8): 1111-1122.*

*4(b). Harrison, D., Muskett, H., Harvey, S., Grieve, R., Shahin, J., Patel, K., Sadique, Z., Allen, E., Dybowski, R., Jit, M., Edgeworth, J., Kibbler, C., Barnes, R., Soni, N., Rowan, K.M. (2013), Development and validation of a risk model for identification of non-neutropenic, critically ill adult patients at high risk of invasive Candida infection: the Fungal Infection Risk Evaluation (FIRE) Study, Health Technology Assessment, 17(3):1-156.*
